# Amyloidogenicity and toxicity of the reverse and scrambled variants of amyloid‐β 1‐42

**DOI:** 10.1002/1873-3468.12590

**Published:** 2017-02-28

**Authors:** Devkee M. Vadukul, Oyinkansola Gbajumo, Karen E. Marshall, Louise C. Serpell

**Affiliations:** ^1^ School of Life SciencesUniversity of SussexFalmerUK

**Keywords:** Alzheimer's disease, amyloid fibril, amyloid‐β, control, cytotoxicity, self‐assembly

## Abstract

β‐amyloid 1‐42 (Aβ1‐42) is a self‐assembling peptide that goes through many conformational and morphological changes before forming the fibrils that are deposited in extracellular plaques characteristic of Alzheimer's disease. The link between Aβ1‐42 structure and toxicity is of major interest, in particular, the neurotoxic potential of oligomeric species. Many studies utilise reversed (Aβ42‐1) and scrambled (AβS) forms of amyloid‐β as control peptides. Here, using circular dichroism, thioflavin T fluorescence and transmission electron microscopy, we reveal that both control peptides self‐assemble to form fibres within 24 h. However, oligomeric Aβ reduces cell survival of hippocampal neurons, while Aβ42‐1 and Aβs have reduced effect on cellular health, which may arise from their ability to assemble rapidly to form protofibrils and fibrils.

## Abbreviations

**AD**, Alzheimer's disease

**CD**, circular dichroism

**HBSS**, Hank's Balanced Salt Solution

**HFIP**, hexafluoro‐2‐isopropanol

**TEM**, transmission electron microscopy

**ThT**, thioflavin T fluorescence

A key characteristic of Alzheimer's disease (AD) is the deposition of β‐amyloid fibrils in extracellular plaques, as well as the intracellular accumulation of tau in neurofibrillary tangles in the brain 1. Whether Aβ or tau is the trigger or driver of the disease continues to be a controversial topic, although there is no doubt that both contribute to the disease pathway and progression 2. Aβ is cleaved from the amyloid precursor protein (APP) to produce several peptides of different amino acid‐length peptides including 1‐39, 1‐40, 1‐42, 1‐43 and 1‐46 as well as the N‐truncated Aβ4‐42 3 While Aβ1‐40 is the predominant species in unaffected individuals, the ratio of Aβ1‐42:1‐40 increases in AD 4. Aβ1‐42 is more amyloidogenic than Aβ1‐40 *in vitro* 5 and appears to show a higher level of toxicity in cellular assays 6. Furthermore, increased levels of Aβ1‐42 correlate with Alzheimer's disease in both familial and sporadic Alzheimer's disease patients 7. Therefore, the self‐assembly of Aβ1‐42 is implicated in the cause of AD.

The cytotoxic effect of the Aβ1‐42 peptide is believed to be linked to its ability to self‐assemble to form oligomers and amyloid fibrils 8. This is supported by our recent report showing that a designed nonassembling variant of Aβ1‐42 is unable to induce cell death of hippocampal neurons 9. The oligomeric form is proposed to represent the toxic species leading to neuronal dysfunction and eventual cell death 8, 10. Many studies have examined the role of Aβ1‐42 and have utilised Aβ42‐1 or AβS as control peptides 11, 12_._ These “control” peptides are chosen because they are not expected to self‐assemble or to form toxic, oligomeric species, despite sharing amino acid composition with wild‐type Aβ. However, the fibrillogenesis of these peptides and resulting structures has not been previously analysed in detail. Furthermore, despite being used in cellular assays as controls, the cytotoxic nature, or lack of, has not been studied in relation to their assembly state. Here, we have characterised the assembly, structure and toxicity of the current experimental controls used in Aβ1‐42 studies; Aβ42‐1 and AβS. Understanding the biophysical properties of these peptides provides valuable information about the ability of peptides to form toxic species and gives further insights into how sequence relates to amyloidogenicity and/or toxicity.

We have optimised a preparation process for the Aβ1‐42 peptide allowing us to follow aggregation from monomer to fibril consistently 8, 13. Pretreatment of Aβ1‐42 is essential as self‐assembly is extremely difficult to control due to the peptides’ sensitivity to temperature, pH, concentration and the presence of pre‐existing aggregates which can act as seeds and accelerate assembly. Using this information, we are able to confidently identify the time point where Aβ1‐42 oligomers are most abundant in solution, and use this information to assess the relationship between structure and toxicity.

Here, Aβ1‐42, Aβ42‐1 and AβS were prepared in an identical manner and their fibrillogenesis and structure were examined using circular dichroism (CD), thioflavin T fluorescence (ThT) and transmission electron microscopy (TEM). We show that both control peptides adopt β‐sheet structure earlier than Aβ1‐42_,_ and also aggregate more rapidly to form mature fibrils. The toxic effect of these peptides was investigated on primary hippocampal neuronal cultures; as expected, Aβ1‐42 oligomers showed significant toxicity. In contrast, the two control peptides exerted minimal cytotoxicity. This may arise from the formation of elongated, fibrillar structures at the time of administration to cells.

## Methods

### Preparation of peptides

Aβ42‐1 and AβS (Table 1) were purchased from Bachem in powder form with free N and C termini. (AβS sequence can differ depending on company – this sequence is sold by Anaspec and BACHEM but scrambled sourced from rPeptide and Biolegend differ in sequence). 1,1,1,3,3,3‐hexafluoro‐2‐isopropanol (HFIP) films of recombinant Aβ1‐42 were purchased from rPeptide. Peptides were prepared in the appropriate buffer depending on the experimental procedure being used. About 0.2 mg of peptide was solubilised in 200 μL HFIP (Sigma Aldrich, Dorset, UK) in order to remove any preaggregates. The solution was then vortexed for a minute and sonicated for 5 min in a 50/60 Hz sonicator. Nitrogen gas was used to evaporate the HFIP, after which 200 μL dry dimethylsulphoxide (DMSO) (Sigma‐Aldrich) was added. The solution was again vortexed for a minute, followed by sonication for a minute. The solutions were added to buffer‐exchange, HEPES‐equilibrated 7KDMW Zeba columns with 40 μL buffer added as a stack. The protein solution collected was kept on ice and the absorbance at 280 nm was measured immediately with a NanoDrop spectrophotometer using a molar coefficient of 1490 m ^−1^ cm^−1^ (value from http://web.expasy.org/protparam/). All solutions were diluted to 50 μm with buffer.

**Table 1:** Peptide sequences

Peptide	Sequence
Wild‐type Aβ1‐42	DAEFRHDSGYEVHHQKLVFFAEDVGSNKGAIIGLMVGGVVIA
Scrambled Aβ1‐42s	AIAEGDSHVLKEGAYMEIFDVQGHVFGGKIFRVVDLGSHNVA
(Reversed) Aβ42‐1	AIVVGGVMLGIIAGKNSGVAGAFFVLKQHHVEYGSDHRFEAD

### Waltz algorithm analysis

The primary sequence of each peptide (Table 1) was input in FASTA format into the Waltz algorithm http://waltz.switchlab.org and pH was set to 7 14. Data were output as a text file (.dat) and then plotted using excel.

### Transmission electron microscopy

Peptides were prepared as described above in 20 mm phosphate buffer pH 7.4 (200 mm Na_2_HPO_4_, 200 mm NaH_2_PO_4_ diluted to 20 mm with ddH_2_O). Aliquots of the peptides were taken at 2, 4 and 24 h to assess the progression of fibrillisation and morphology. 4 μL of 50 μm peptide solution was placed on Formvar/carbon film‐coated, 400‐mesh copper grids (Agar Scientific), allowed to absorb for 1 min and blotted dry. The grid was washed with 4 μL 0.2 μm milliQ‐filtered water and blotted dry after which 4 μL 2% (w/v) uranyl acetate was added to the grid for 1 min. The dye was then blotted dry and the grid was left to air dry. All grids were examined using a JEOL JEM1400‐Plus TEM at 120 kV and the images were captured using a Gatan OneView 4K camera (Abingdon, UK).

### Circular dichroism

150 μL of 50 μm peptide solution was prepared as described above in 20 mm phosphate buffer. A 1‐mm pathway cuvette (Hellma, Essex, UK) was used for Aβ1‐42 and Aβ42‐1. A 0.5‐mm (Hellma) cuvette was used for AβS. Scans were taken at a scanning speed of 100 nm·min^−1^, using a slit width of 1 μm taken between 180 nm and 320 nm on a JASCO (Essex, UK) J715 Spectropolarimeter. A water bath was used to equilibrate samples at 20 °C and the average of three spectra was used for each measurement. Spectral data were converted to molar ellipticity using the following equation: Mdeg × Molecular weight/(10 × mg·ml^−1^ × pathlength of cuvette × number of amino acids).

### Thioflavin T fluorescence

Thioflavin T (10 μm) in 50 μm peptide solution prepared in 20 mm phosphate buffer was added to a 10‐mm cuvette. A Varian Cary Eclipse Fluorescence Spectrophotometer was used to perform an emission scan between 460 and 600 nm. Excitation and emission slits were set to 5 and 10 nm respectively. The sample compartment was kept at a temperature of 21 °C, scan rate was 600 nm·min^−1^ and the average of three spectra was used for each measurement.

### Cell culture

Rats were housed within a specialised facility under Home office guidelines and sacrificed using procedures in accordance with Animals (Scientific Procedures) Act 1986, Amendment Regulations 2012 and with local ethics approval (University of Sussex Ethical Research committee). Primary neurons were prepared from P0‐1 rats initially by dissecting tissue into ice cold Hank's balanced salt solution (HBSS) containing 0.1 m HEPES. Following washes in prewarmed Basal Medium Eagle (Gibco, Waltham, MA, USA) containing 0.5% glucose, 2% FCS, 1 mm sodium‐pyruvate, 0.01 m HEPES pH 7.35, 1% penicillin–streptomycin, 1% B27 supplement and 1% Glutamax, tissues were triturated using a 1 mL pipette until fully dissociated. The cell suspension was diluted further with complete Basal Medium Eagle media and approximately 40 000 cells were plated into 2‐cm^2^ wells containing a coverslip coated in 20 μg·mL^−1^ Poly‐D‐Lysine with a layer of hippocampal astrocytes that had been growing for 4–5 days. After 2–3 days, cells were treated with 3.25 μm cytosine arabinoside to halt further proliferation of astrocytes. Cells were used 10–14 days after plating.

### Cell viability assay with primary hippocampal neurons

Peptides were prepared in 10 mm HEPES buffer (10 mm HEPES, 50 mm NaCl, 1.6 mm KCL, 2 mm MgCl_2_, 3.5 mm CaCl_2_). After incubation with the peptide for the desired time, one drop of each Readyprobe reagent (Life Technologies, Waltham, MA, USA) was added to each well. The blue stain reagent is used to label all cells and the green stain reagent is to label only the dead cells. The cells were incubated with the reagents for the required 15 min before being imaged on a Zeiss (Cambridge, UK) CO widefield microscope using DAPI and FITC filters. Analysis was carried out using FIJI software to calculate the percentage of blue cells that were also stained green. Cells were counted using the cell counter plug‐in and astrocytes were excluded in the counting. Two coverslips per sample were used and at least four regions of interest were imaged from each. The experiment was repeated three independent times.

## Results and Discussion

### Aβ42‐1 and AβS assemble to form mature fibrils with β‐sheet structure

**Figure 1:**
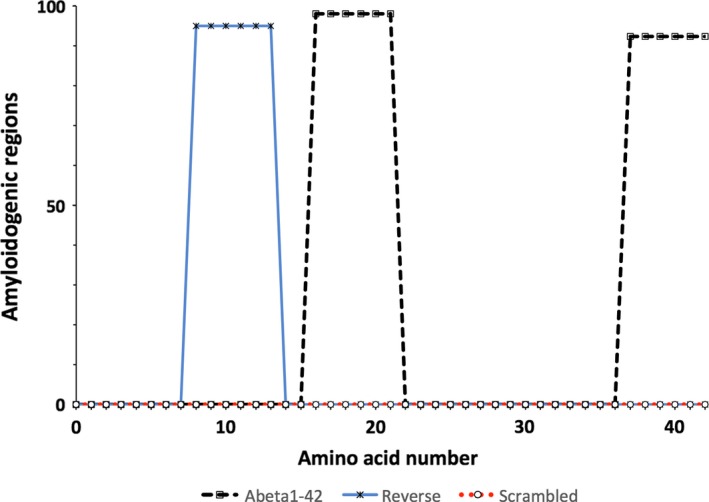
Graphs produced using WALTZ 14 for Aβ1‐42, Aβ42‐1 and AβS. There are two amyloidogenic regions identified for Aβ1‐42 between residues 16‐21 and 37‐42, one region was identified for Aβ42‐1 between residues 8 and 13 and no amyloidogenic regions were identified for AβS.

WALTZ algorithm, which identifies amyloidogenic regions using a positional algorithm, was used to predict the peptides propensity to aggregate 14. The graphical trace produced for Aβ1‐42 highlights two amyloidogenic regions, while a single region is predicted for Aβ42‐1 and AβS is predicted to have no amyloidogenic regions (Fig. 1).

Using a range of biophysical techniques, the assembly and structure of the two control peptides were monitored and compared to the wild‐type peptide (Table 1). Peptides were pretreated with hexafluoro‐2‐isopropanol (HFIP) and DMSO followed by buffer‐exchange using a spin column to ensure that any preaggregates and remaining solvents were removed. Following this, the stock solution of each peptide was immediately diluted to 50 μm in buffer to ensure consistency and reproducibility between experiments as changes in concentration can significantly affect assembly. The assembly of the peptides was monitored over a 7‐day period using TEM, CD and ThT fluorescence.

**Figure 2:**
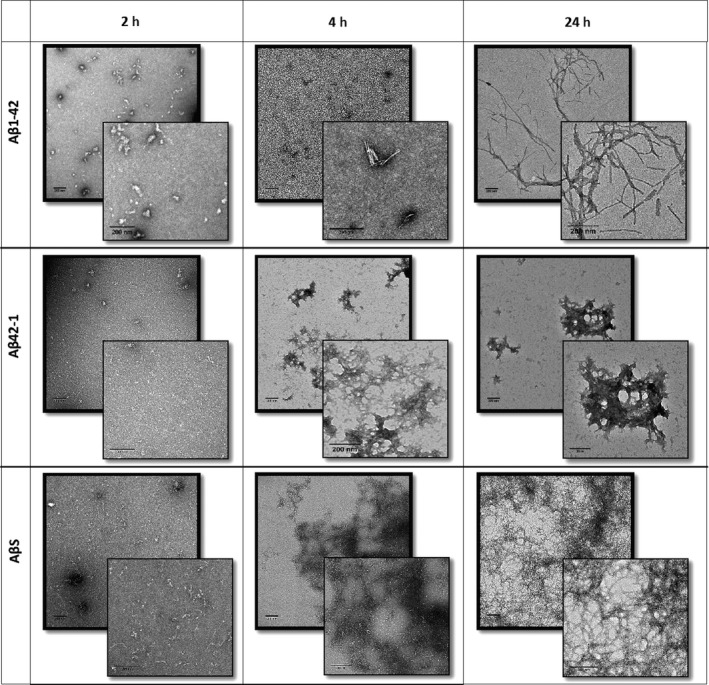
Negative stain transmission electron micrographs with magnified images. Aβ1‐42 (top row), Aβ42‐1 (middle row) and AβS (bottom row) at 2, 4 and 24 h show Aβ1‐42 assembly from small spherical oligomers to long fibrils by 24 h. Aβ42‐1 shows slightly larger spherical species at 2 h which form clumps of fibrils by 4 h. AβS shows small fibrils by 2 h and fibrillar networks by 4 h which are larger by 24 h. Scale bars 200 nm.

Electron microscopy was used to observe the morphology of peptide assemblies over time (Fig. 2). It is evident that by 24 h, Aβ42‐1 forms fibrillar structures though these are less ordered in appearance compared to those formed by Aβ1‐42. Interestingly, AβS forms plaque‐like fibrillar networks by 24 h which are only observed with Aβ1‐42 after 7 days at the same concentration 9. This suggests that not only does AβS aggregate, but also does so with a higher propensity than Aβ1‐42_._ This result was unexpected as WALTZ predicted no amyloidogenic regions for AβS. This highlights the importance of conducting experimental structural characterisation. Despite WALTZ being an excellent tool in predicting peptide amyloidogenic regions, AβS does assemble which suggests the amyloidogenic nature of a peptide is more complex than primary sequence alone.

**Figure 3:**
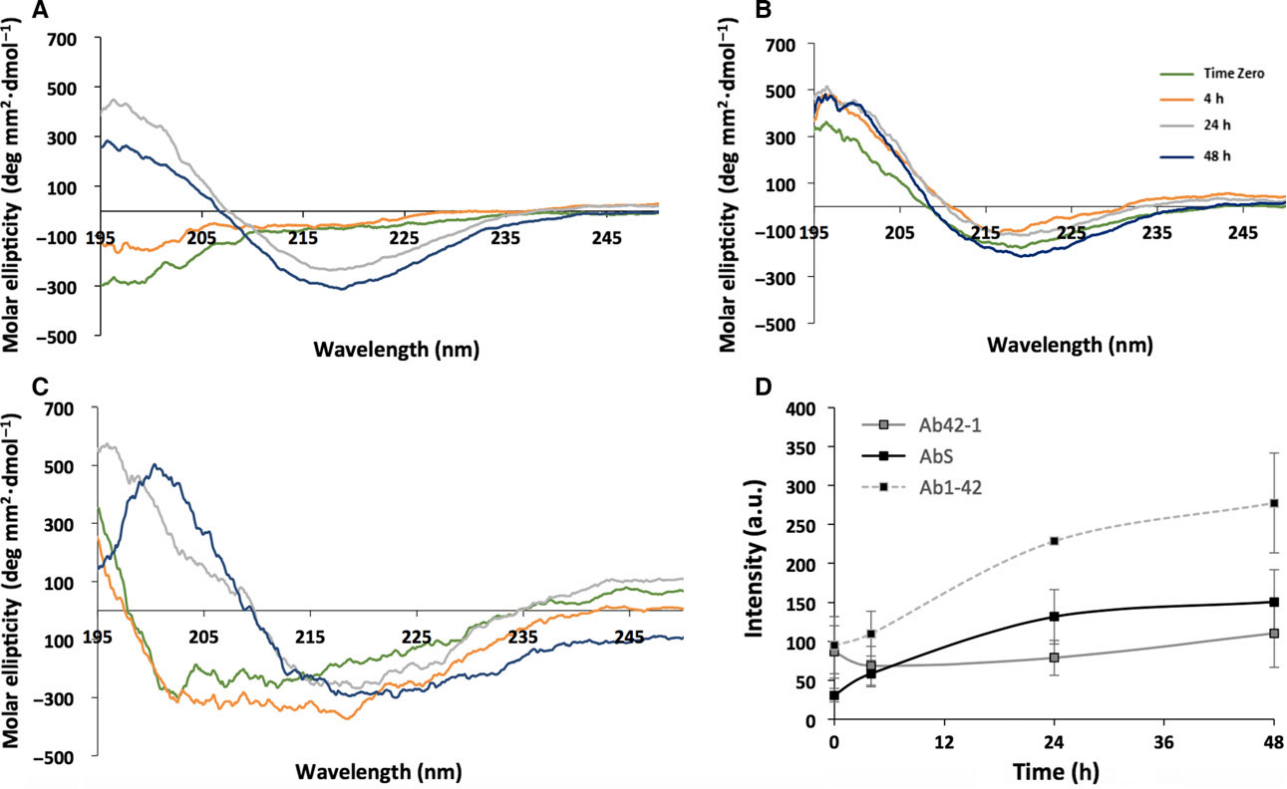
(A–C) CD spectra of (A) Aβ1‐42, (B) Aβ42‐1 and (C) AβS respectively. The spectrum for Aβ1‐42 shows the formation of β‐sheet structure at 24 h, while both Aβ42‐1 and AβS display spectra for β‐sheet structures from T0. (D) ThT fluorescence over time for Aβ1‐42 (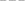), Aβ42‐1 (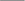) and AβS (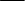). All peptides displayed fluorescence at 483 nm. Aβ1‐42 shows a lag phase before an increase in intensity, which then plateaus. Aβ42‐1 shows a very slight increase in intensity over time and AβS has no lag phase but an increase in intensity over time, which also plateaus over time. An average of three experiments is shown.

Circular dichroism was used to investigate the secondary structure of each peptide over the incubation time (Fig. 3A–C). CD spectrum from Aβ1‐42 demonstrates a conformational change from a random coil (trough centred at 190 nm) to β‐sheet structure (trough and peak centred at 218 nm and 192 nm respectively) from 0 to 24 h as previously described 9. In comparison, both control peptides show a strong β‐sheet signal almost immediately after preparation. The spectrum for AβS is initially shifted slightly towards random coil, suggesting a mixed population, but displays a strong β‐sheet signal by 24 h. These data indicate that the Aβ42‐1 and AβS peptides form initial β‐sheet structure rapidly following preparation, while the wild‐type protein transitions from random coil to β‐sheet over a 24‐h period under the conditions used.

Fibrillogenesis was monitored using a ThT fluorescence assay (Fig. 3D); both control peptides show a signal at 483 nm, which indicates the presence of ThT‐positive structures. The spectrum for wild‐type Aβ shows a lag phase before a steep increase in intensity, which then begins to plateau after 20 h of incubation. This can be explained by nucleation‐dependent fibrillisation; the lag phase is the period during which oligomers are generated through a primary pathway and act as thermodynamically stable nuclei for fibril growth. Once a critical fibril concentration has been reached, primary nucleation is overtaken by secondary nucleation and the surface of these fibrils can act as a catalyst for the formation of oligomers and further proliferation into fibrils 15. This lag and elongation phase is not observed with either of the control peptides. This is consistent with the results from TEM and CD which suggest that the peptides assemble very rapidly after preparation. The lower intensity in ThT fluorescence at later time points may be attributed to the loss of peptide from the solution as larger aggregates form and this is supported by the dense fibril morphology observed in the electron micrographs at 24 h. The ThT fluorescence spectrum for Aβ42‐1 showed a very shallow increase in intensity, although fibrils are present in the electron micrographs. Although different peptide systems are difficult to directly compare using ThT due to differential binding, it is clear that the ThT intensity increases with incubation time, suggesting that all three peptides self‐assemble to form amyloid fibrils during the timeframe of the experiment.

To compare the molecular structures of the fibrils formed by the wild‐type and variant peptides, X‐ray fibre diffraction patterns were collected. The fibrils were partially aligned and the X‐ray fibre diffraction patterns revealed characteristic cross‐β diffraction signals at 4.7 Å and at 10 Å (Fig. S1) consistent with characteristic cross‐β structure for amyloid 16, 17. Unfortunately, the alignment of the samples is insufficient for detailed conclusions to be drawn regarding structural similarities or differences between the fibrils formed by the peptides.

In conclusion, CD and ThT fluorescence combined with TEM, confirm the presence of β‐sheet structure and fibrillogenesis for all three peptides. Furthermore, a cross‐β pattern from X‐ray fibre diffraction confirms that these control peptides self‐assemble and form bonafide amyloid fibrils.

### Neurotoxicity of Aβ42‐1 and AβS

**Figure 4:**
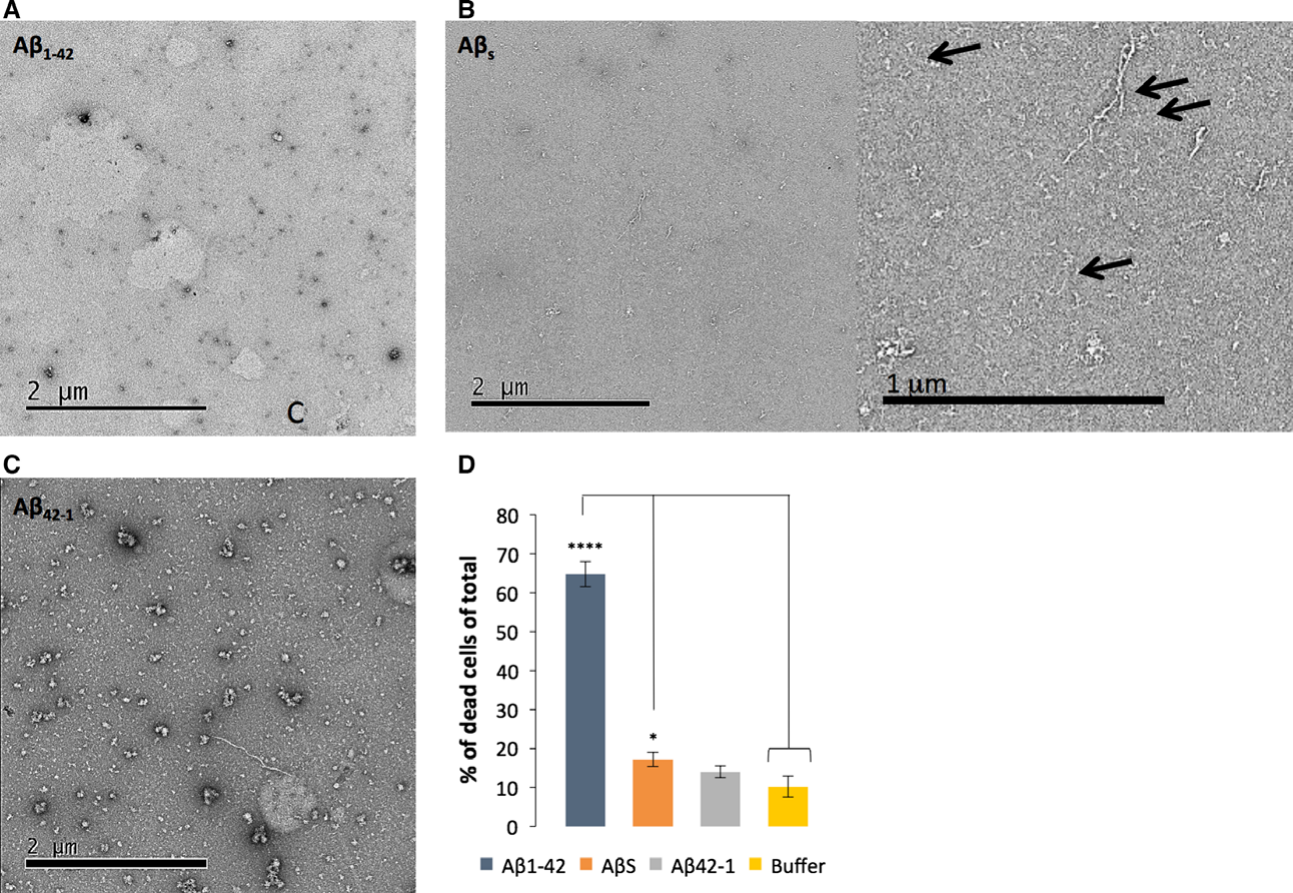
Assessment of cytotoxicity in primary hippocampal cultures treated with peptide at 10 μm and measured using ReadyProbes assay after 7 days of incubation. (A–C) Electron micrographs of the peptides added to cultures after 2‐h incubation. Scale bars and shown. A magnified image is shown for AβS (B) and arrows point to protofibrillar structures. (D) ReadyProbes assay was conducted after 7 days to assess toxicity relative to the buffer control (*n* = 558, dead cells = 52) (*P* = < 0.05 (*), < 0.01 (**), < 0.0001 (****) and > 0.05 = not significant). Aβ1‐42 showed significant cytotoxicity (*n* = 752, dead cells = 490), Aβ42‐1 showed no significant cytotoxicity (*n* = 719, dead cells = 97) and AβS showed minimal significant cytotoxicity (*n* = 1627, dead cells = 288).

As described above, oligomeric Aβ1‐42 is thought to be the toxic species 10, 18, 19. In order to investigate and compare the potential cytotoxicity of the self‐assembled peptides, a ReadyProbes cell viability assay (Life Technologies) was conducted. A blue reagent stain was used to label all cells, while a green reagent stain labels dead cells only. The percentage of dead cells in the entire cell population was calculated as a measure of toxicity. It is important to note that although cell viability assays are widely used to investigate cytotoxicity, the relationship to AD pathology may not be closely linked due to the various different pathways involved in cell death and the specificity of the assay being used 20. Nevertheless, as we are directly investigating the cytotoxicity of these control peptides in hippocampal neurons and not modelling AD pathology, we believe the ReadyProbes assay to be a valid approach. Previously, we developed a method to prepare oligomeric Aβ1‐42 by freshly preparing the peptide, diluting the stock to 50 μm and incubating it at room temperature for 2 h before adding to rat hippocampal neurons 9. The cell death assay was then conducted after 7 days of incubation in the presence of the peptide. Here, identical methods of preparation were used for both control peptides and compared to the hippocampal neurons incubated with wild‐type Aβ1‐42. Electron micrographs were also prepared in order to visualise morphologies of the peptides at the time point at which the peptide was added to the neurons (Fig. 4A‐C).

Aβ1‐42 generally causes 65% (SEM ± 3.24, ‘****’) cytotoxicity after 7 days of incubation compared to the buffer only condition that showed only 10% cell death (SEM ± 2.75) (Fig. 4D). In comparison, AβS and Aβ42‐1 demonstrated less potent toxicity than wild‐type peptide at the same time point and using the identical preparation conditions; 17% (SEM ± 1.55,‘*’) and 14% (SEM ± 1.86) respectively. Previous studies have also shown that Aβ42‐1 21 and AβS (identical sequence to AβS used here from Anachem) 11 are inactive in cellular assays. Electron micrographs taken 2 h after preparation offer an explanation for the difference in cytotoxicity of these peptides. The wild‐type Aβ1‐42 shows small spherical species, which we identify as oligomers. In contrast, electron micrographs of Aβ42‐1 and AβS taken at the same time point show some larger aggregates, protofibrils and some fibrils. It appears that reverse and scrambled variants of Aβ1‐42 self‐assemble to form fibrillar structures more rapidly than wild‐type and these are much less toxic than the oligomeric wild‐type peptide. Previous work has linked internalisation of oligomers with toxicity and therefore one hypothesis for the reduced toxic nature of these peptides is that these structures are less able to enter the neurons 9, 22 and cause their downstream toxic effects due to their increased size. The inability for cells to take up AβS has been previously reported 23, as has the nonapoptotic effects 24 and Aβ42‐1 25. This supports the view that toxicity of amyloidogenic proteins is tightly linked to assembly size and structure. It has previously been reported that the uptake of aggregating amyloid proteins is sequence specific 26, and the cellular response to these proteins is thought to be highly dependent on aggregation propensity, size and charge. As the sequences of both control peptides have led to a higher propensity to aggregate than wild‐type Aβ1‐42, confirmed by structural characterisation presented above, the reported mechanisms of internalisation, which include dynamin‐mediated endocytosis 27, may not be possible with the control peptides. Alternatively, the specific order of amino acids in Aβ42‐1 and AβS may affect binding to receptors or assembly into specific oligomeric species that are required for toxicity.

## Conclusions

Although both Aβ42‐1 and AβS have been used in many studies as experimental controls for Aβ1‐42_,_ these results suggest their validity should be questioned. If conclusions are to be made regarding the effects of amyloidogenic proteins, it is desirable for controls to be sequence related but ideally show no propensity for self‐assembly. We have previously presented a sequence‐related, nonaggregating variant of Aβ1‐42 9 as a comparison to these traditional controls and also as an example of a more suitable control. To critically evaluate and continually develop our experimental controls in this way will ultimately aid our understanding of the mechanisms involved in AD. Furthermore, by characterising assembly and relating this to toxicity, our findings suggest that the toxic nature of these amyloid proteins is likely to be related to size and sequence. WALTZ predicted both control peptides to have fewer amyoidogenic regions than the wild‐type; Aβ42‐1 was predicted to have one amyloidogenic region, whereas AβS was predicted to have none. Despite this, both peptides show assembly and similar noncytotoxic behaviour in cells. We suggest that this is due to the lack of oligomeric species formed by both control peptides, which supports the view that Aβ1‐42 oligomers represent the toxic entity.

## Author contribution

DV and OG conducted the structural experiments. DV conducted cellular experiments. KM and LCS planned the experiments and managed the work. DV and LCS wrote the manuscript and KM edited the manuscript.

## Supporting information


**Fig. S1.** X‐ray fibre diffraction patterns for partially aligned fibrils formed by Aβ42‐1 and AβS.
