# Supplementary material for: Amyloidogenicity and toxicity of the reverse and scrambled variants of amyloid‐β 1‐42
Source: FEBS Lett. 2017 Feb 28;591(5):822–30. doi: 10.1002/1873-3468.12590 (PMC5363225; doi:10.1002/1873-3468.12590)
Supplement: Supplementary file 1 — Fig. S1. X‐ray fibre diffraction patterns for partially aligned fibrils formed by Aβ42‐1 and AβS. [file FEB2-591-822-s001.pdf]

Supplementary information

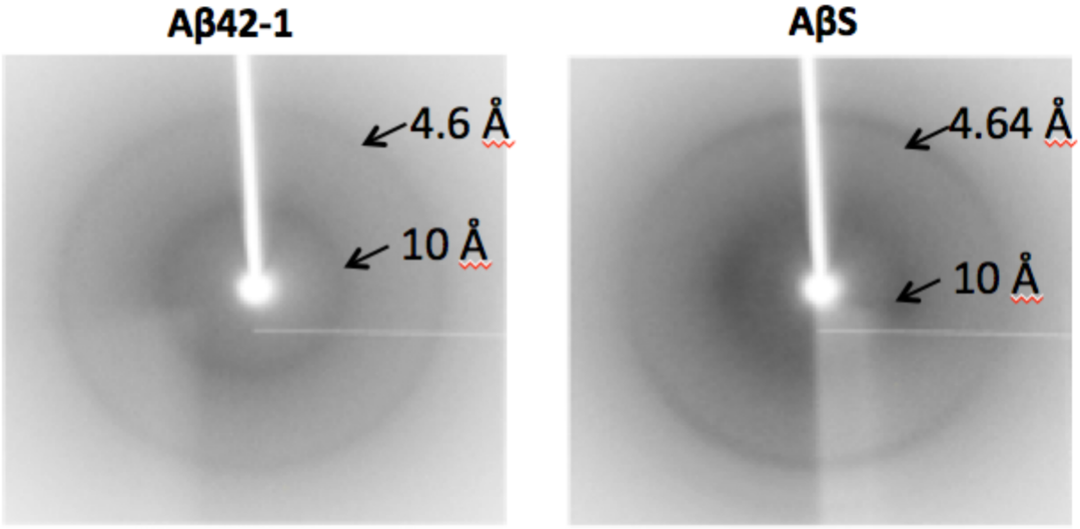

Figure S1. X-ray fibre diffraction patterns for partially aligned fibrils formed by Aβ42-1 and AβS. X-ray fibre diffraction patterns revealed characteristic cross-β diffraction signals at 4.7 Å and at 10 Å.
